# Next-Generation Liver Medicine Using Organoid Models

**DOI:** 10.3389/fcell.2019.00345

**Published:** 2019-12-20

**Authors:** Soheil Akbari, Nur Arslan, Serif Senturk, Esra Erdal

**Affiliations:** ^1^İzmir Biomedicine and Genome Center, İzmir, Turkey; ^2^Department of Pediatric Gastroenterology and Metabolism, Faculty of Medicine, Dokuz Eylul University, İzmir, Turkey; ^3^Department of Genome Sciences and Molecular Biotechnology, İzmir International Biomedicine and Genome Institute, Dokuz Eylul University, İzmir, Turkey; ^4^Department of Medical Biology and Genetics, Faculty of Medicine, Dokuz Eylul University, İzmir, Turkey

**Keywords:** organoids, liver, disease modeling, adult stem cells, iPSCs, 3D cell culture systems

## Abstract

“*Liver medicine*” refers to all diagnostic and treatment strategies of diseases and conditions that cause liver failure directly or indirectly. Despite significant advances in the field of liver medicine in recent years, improved tools are needed to efficiently define the pathophysiology of liver diseases and provide effective therapeutic options to patients. Recently, organoid technology has been established as the state-of-the-art cell culture tool for studying human biology in health and disease. In general, organoids are simplified three-dimensional (3D) mini-organ structures that can be grown in a 3D matrix where the structural and functional aspects of real organs are efficiently recapitulated. The generation of organoids is facilitated by exogenous factors that regulate multiple signaling pathways and promote the self-renewal, proliferation, and differentiation of the cells to promote spontaneous self-organization and tissue-specific organogenesis. Newly established protocols suggest that liver-specific organoids can be derived from either pluripotent stem cells or liver-specific stem/progenitor cells. Today, robust and long-term cultures of organoids with the closest physiology to *in vivo* liver, in terms of cellular composition and function, open a new era in studying and understanding the disease pathology as well as high-throughput drug screening. Of note, these next-generation cell culture systems have immense potential to be further improved by genome editing and bioengineering technologies to foster the development of patient-specific therapeutic options for clinical applications. Here, we will discuss recent advances and challenges in the generation of human liver organoids and highlight emerging concepts for their potential applications in liver medicine.

## Introduction

The prevalence of liver diseases is rising, and they account for approximately 2 million deaths per year worldwide (Asrani et al., 2019). The etiologies of chronic liver diseases are multifactorial and show variation according to geographical location (Zhou et al., 2014). The main causes include chronic viral infections (hepatitis B and C), excessive alcohol intake, obesity-related fatty liver disease and steatohepatitis, inherited diseases (Wilson’s disease, storage disorders, hepatorenal tyrosinemia, etc.), autoimmune liver diseases, drug-related liver diseases, as well as malignancies and idiopathic causes (Zhou et al., 2014).

To date, various cell culture and animal models have been used to decipher the molecular mechanisms of liver development and pathogenesis. Among them, conventional two-dimensional (2D) cell cultures have several limitations, especially for long-term and stable expansion. In addition, 2D primary hepatocyte cultures fail to replicate key aspects of the human liver tissue, in particular, the complex architecture and cellular heterogeneity (Duval et al., 2017; Rowe and Daley, 2019). Moreover, these cells often lack cell–cell and cell–extracellular matrix (ECM) interactions that are essential for maintaining *in situ* phenotypes and biological functions as well as tissue-specific cellular processes (Baxter et al., 2015; Duval et al., 2017). Furthermore, primary hepatocytes have limited division capacity when grown in 2D cultures and rapidly lose liver-specific gene expression patterns and functions, such as the synthesis of coagulation inhibitors (Boost et al., 2007), maintenance of stable cytochrome P450 (Darnell et al., 2012; Kostadinova et al., 2013), and integrin ligation (Meli et al., 2012), at a few weeks after plating (Clayton and Darnell, 1983; Bissell et al., 1987; Mitaka and Ooe, 2010).

Ever since induced pluripotent stem cell (iPSC) technology was established, hepatocyte generation by stepwise differentiation protocols that mimic *in vivo* organogenesis has become readily feasible in 2D cultures (Takahashi et al., 2007; Si-Tayeb et al., 2010b; Chen et al., 2012; Hannan et al., 2013). These protocols typically use cocktails of growth factors/cytokines in order to recapitulate embryonic liver development under *in vitro* culture conditions. Nevertheless, the hepatocytes derived by these differentiation protocols vary considerably in their maturation level and, in most cases, represent immature hepatocytes. Plus, they do not have the ability to expand for the long term in monolayer culture, partly due to the absence of a tissue-specific architecture, mechanical and biochemical cues, and cell–cell communications under 2D conditions (Pampaloni et al., 2007; Luo et al., 2018).

Animal models can also be used in the study of liver pathologies. The large majority of these models have functional vasculature, stromal, and immune components, offering numerous benefits over 2D cultures. However, animal models need resource-intensive and time-consuming processes to be developed. Furthermore, physiological and genomic interspecies differences in animal models pose limitations in the representation of the disease phenotypes and the prediction of research outputs such as drug response (Mariotti et al., 2018).

Organoids are simply defined as 3D cell culture systems that mimic the structural and functional characteristics of the represented organ. This definition refers to the assembly of organ-specific cell type(s) that develop from pluripotent stem cells/organ stem/progenitor cells and their self-organization through cell sorting and spatially restricted lineage commitment, similar to the developmental process *in vivo* (Lancaster and Knoblich, 2014; Clevers, 2016; Fatehullah et al., 2016). Organoid structures can be stably maintained in long-term *in vitro* cultures, continuing to reflect, even after many generations, the *in vivo* characteristics of the tissue of origin without any significant genetic or physiological changes (Huch et al., 2015; Akbari et al., 2019). During the process of organoid culture, often in a tissue-specific manner, a number of growth factors and small molecules are utilized in order to regulate the signaling pathways that are essential for the self-renewal, differentiation, and proliferation of organoids. At present, two main approaches to successfully forming organoids using defined biochemical factors in a proper 3D matrix have been widely exploited. The first approach is based on the differentiation of pluripotent stem cells (PSCs), which then self-organize to form tissue-specific organoids such as the optic cup (Eiraku et al., 2011; Nakano et al., 2012; Kuwahara et al., 2015; Eldred et al., 2018), brain (Lancaster et al., 2013; Pas̨ca et al., 2015), intestine (Spence et al., 2011), pancreas (Greggio et al., 2013), liver (Takebe et al., 2013; Sampaziotis et al., 2015; Guan et al., 2017; Lee et al., 2018; Akbari et al., 2019; Koike et al., 2019), lung (Dye et al., 2015), prostate (Drost et al., 2016), kidney (Takasato et al., 2015), and blood vessel (Wimmer et al., 2019). The second approach relies on the derivation of functional organoids from tissue-specific adult/fetal/pediatric stem or progenitor cells, which preserve, under normal and damaged conditions, the regenerative capacity of specific tissues. These types of organoids have also been established from multiple tissues including intestine (Ootani et al., 2009; Sato et al., 2009), stomach (Barker et al., 2010), liver (Huch et al., 2015; Hu et al., 2018), kidney (Schutgens et al., 2019), skin (Boonekamp et al., 2019), and pancreas (Huch et al., 2013a; Boj et al., 2015).

## Liver

### Structure and Function

The liver, the largest organ in the body, serves vital metabolic, exocrine, and endocrine functions. In addition to bile production and regulation of glycolytic, urea, and cholesterol metabolism, the liver promotes blood detoxification and regulates blood homeostasis, particularly by secreting coagulation factors and serum proteins such as albumin (Gordillo et al., 2015; Stanger, 2015). The liver lobule, composed of heterogeneous cell types, is the smallest structural unit of the liver. Metabolically, it is divided into different structural zones and functional organizations (Kietzmann, 2017). Hepatocytes are the major parenchymal cell type of the liver lobules, accounting for ∼70% of the total mass of an adult organ. Hepatocytes and cholangiocytes (the other epithelial cell type of the liver) are derived from the embryonic endoderm, while the non-parenchymal liver cells, such as the stromal cells, stellate cells, Kupffer cells, and blood vessels, have a mesodermal origin (Gordillo et al., 2015; Stanger, 2015). Recent advanced technologies, in particular, single-cell RNA sequencing, have made it possible to identify the existence of adult human liver-resident epithelial progenitors and to map stem/progenitor heterogeneity within the liver. These new insights have provided strong guidance cues for advancing understanding of liver biology and physiology (Aizarani et al., 2019).

### Liver Organogenesis

The main principle of liver organoid technology is to recapitulate the major phases of organogenesis in a dish. An understanding of sequential events and regulatory factors in liver development is therefore crucial (Tremblay and Zaret, 2005; Si-Tayeb et al., 2010a; Fiorotto et al., 2018). During gastrulation, blastula first gives rise to endoderm (one of the three primary germ layers), which is further patterned into discrete organ domains in the foregut, midgut, and hindgut. The liver bud is then formed from the foregut endoderm by the induction of Activin/Nodal, WNT, FGF, and BMP signals (Douarin, 1975; Gualdi et al., 1996; Jung et al., 1999; Kamiya et al., 1999; Rossi et al., 2001; Zaret, 2002; D’Amour et al., 2005; Han et al., 2011). Following the formation of this structure, the hepatoblasts undergo expansion and differentiation to yield both hepatocytes and biliary epithelium, while the adjacent mesoderm-derived mesenchyme contributes to the generation of liver fibroblasts and stellate cells. The normal structure of the liver tissue is then completed by further maturation of the hepatocytes and cholangiocytes and the cellular integration of the mesenchyme and endothelium (Tremblay and Zaret, 2005).

## Organoid Models

### iPSC-Derived 3D Liver Organoid Models

#### Hepatic Organoids

**FIGURE 1:**
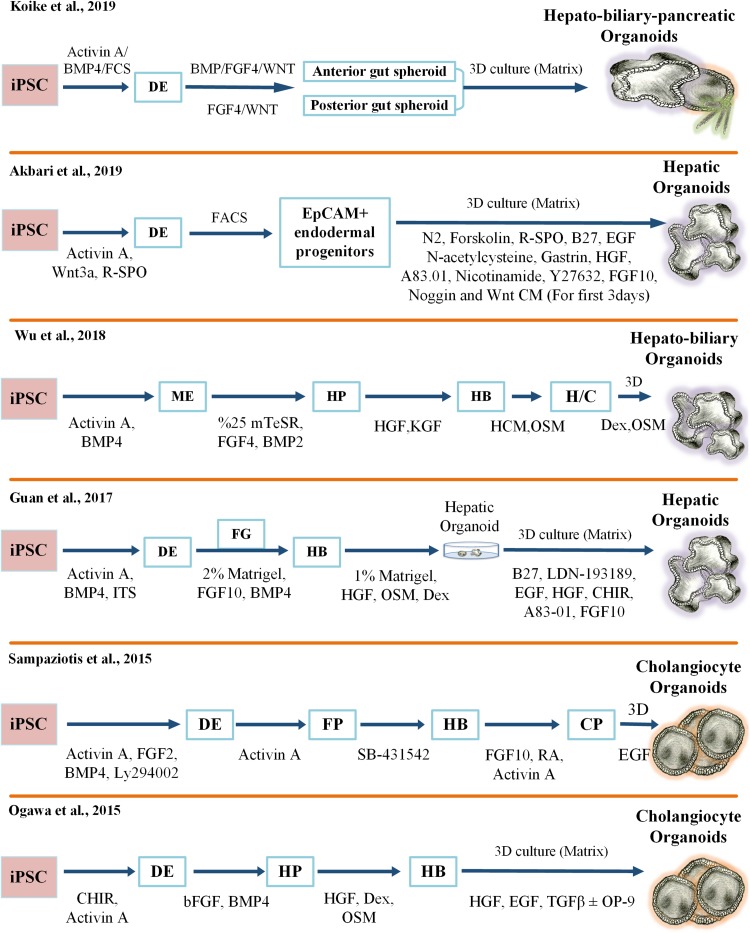
Key protocols for generating iPSC-derived 3D liver organoid cultures. FCS, Fetal Calf Serum; 3D, three-dimensional; FGF, Fibroblast Growth Factor; TGF, Transforming Growth Factor; R-SPO, R-Spondin; EGF, Epidermal Growth Factor; HGF, Hepatocyte Growth Factor; CM, Condition Medium; FACS, Fluorescence-Activated Cell Sorting; EpCAM, Epithelial Cell Adhesion Molecule; iPSC, Induced Pluripotent Stem Cell; BMP, Bone Morphogenetic Protein; ME, Mesoendoderm; HP, Hepatic Progenitor; KGF, Keratinocyte Growth Factor; HB, Hepatoblast; HCM, Hepatocyte Culture Medium; OSM, Oncostatin M; H/C, Hepatocyte/Cholangiocyte; Dex, Dexamethasone; ITS, Insulin-Transferrin-Selenium; DE, Definitive Endoderm; FG, Foregut; FP, Foregut Progenitor; CP, Cholangiocyte Progenitor.

The generation of hepatic organoids from iPCSs was first proposed by Takebe and colleagues using an elegant co-culture model. In this model, hepatic progenitors were obtained by stepwise differentiation from iPSCs in a 2D cell culture setting and then co-cultured with human mesenchymal stem cells (MSCs) and Human Umbilical Vein Endothelial Cells (HUVECs). Subsequently, macroscopically visible 3D aggregates called iPSC-liver buds (LB) were spontaneously produced in a Matrigel-embedded culture. Furthermore, human vasculature structures in iPSC-LB became functional by connecting to the host vessels following transplantation. In particular, the hepatic cells in the engrafted liver buds started to secrete albumin into the bloodstream of the recipient mouse from day 10 until day 45 post-transplantation. More importantly, these organ bud structures demonstrated an ability to regenerate and rescue drug-induced lethal liver failure (Takebe et al., 2013). In a follow-up study, single-cell RNA sequencing elucidated patterns of gene expression unique to lineage identity and facilitated identification of heterogeneous and dynamic cell populations during differentiation from pluripotency to a liver bud (Camp et al., 2017; Potter, 2018). Following these efforts on generating a functional human liver bud, many research groups have developed various protocols to produce different types of liver organoids derived from pluripotent stem cells. Some of the important protocols in the field are summarized in Figure 1.

Guan et al. (2017) have recently established iPSC-derived hepatic organoids (Hep-org), surrounded by cholangiocyte ductal structures, within about 50–60 days. Unfortunately, when these organoids reached a critical size, making it difficult to receive enough nutrients and oxygen, their proliferation and regenerative capacity decreased significantly. This disadvantage was overcome by dissociating the organoids into single cells and then replating and reforming them in Matrigel (Guan et al., 2017). Recently, a new protocol has been described for the generation of human pluripotent stem cell (hPSC)-derived organoids with a hepato-biliary structure. The organoids displayed hepatic gene expression signatures and key functional characteristics of cholangiocytes *in vitro* and survived more than 8 weeks after transplantation in immune-deficient mice (Wu et al., 2019).

More recently, we described a rapid and highly efficient protocol for the production of human hepatic organoids derived from iPSCs. Our study revealed that enrichment of Epithelial Cell Adhesion Molecule (EpCAM)-positive cells resulted in a homogenous population of endodermal cells and licensed the differentiation of functional hepatocytes. In addition, R-Spondin improved the specification of EpCAM-positive endoderm intermediate cells in this culture system (Akbari et al., 2019). Moreover, only EpCAM-positive endodermal cells, and not EpCAM-negative cells, had the ability to form hepatic organoids. Our findings are in strong agreement with previous studies showing that EpCAM exhibited a dynamic pattern of gene expression during liver development and that EpCAM-positive cells were able to give rise to liver compartments (Schmelzer et al., 2007; Tanaka et al., 2009; Lu et al., 2013). In addition, our study showed that these endoderm-derived hepatic organoids (dubbed eHEPOs) could be produced in 14 days and expanded for more than 1 year without any significant loss in culturing efficiency (Akbari et al., 2019). Likewise, *in vitro* characterization analyses indicated that eHEPOs could obtain epithelial morphology and a pseudostratified structure. One of the key aspects of this method was that the cellular composition and the morphological structure of eHEPOs were preserved in young and old organoids. Furthermore, analysis of gene expression profiles revealed a transition from pluripotency toward mature hepatocytes. Finally, eHEPOs exhibited functional characteristics of mature hepatocytes *in vitro*, efficiently engrafted in the mouse liver, and expressed human ALB at day 32 following intrasplenic injection (Akbari et al., 2019). More recently, a breakthrough method has been developed to establish an integral multi-organ structure. First, the anterior and the posterior gut spheroids were separately differentiated from iPSCs. Afterward, an anterior spheroid was transferred to an adjacent posterior spheroid. Over time, these spheres fused and invaginated as an interconnected multi-organ. In sum, that method demonstrated that anteroposterior interaction recapitulated the foregut–midgut boundary *in vitro*, even in the absence of extrinsic factors (Koike et al., 2019).

#### Cholangiocyte Organoids

Another type of organoids that exhibit cholangiocyte characteristics and functions were developed and used by several research groups to specifically study biliary duct physiology and cholangiopathies. For the first time, Dianat et al. (2014) have successfully generated iPSC-derived cholangiocyte-like cells (CLCs) in 3D culture. These cells displayed structural and functional similarity to primary cholangiocytes, such as cilia formation and response to hormonal stimulation. Similarly, CLCs were able to transport Rhodamine 123 into the lumen by the multidrug resistance 1 transmembrane (MDR1), another property of physiologically functional cholangiocytes (Dianat et al., 2014). Then, by combining 2D monolayer and 3D culture systems, Ogawa and colleagues generated cholangiocyte organoids (Chol-org) from hPSCs. This method utilized cholangiocyte development cues to establish a self-organized bile duct-like structure. Cholangiocyte fate was achieved using 3D co-culture of hepatoblasts with OP9 stromal cells in the presence of growth hormones. Herein, NOTCH protein secreted by OP9 cells was used to mimic JAG1/Notch signaling and promote cholangiocyte development and specification. The cholangiocytes exhibited a branching morphology and cholangiocyte-related gene expression profiles. Furthermore, these cells lost the expression of hepatoblast and hepatocyte markers such as Alpha-fetoprotein (AFP) and albumin. Transport activity assay and a forskolin-induced cyst swelling test verified the functionality of these cholangiocytes (Ogawa et al., 2015). Another approach developed by Sampaziotis et al. (2015) demonstrated that Chol-org could be generated from hepatoblast-derived cholangiocyte progenitors using FGF10, Activin A, retinoic acid, and EGF. These Chol-org demonstrated functional properties of cholangiocytes such as alkaline phosphatase and gamma-glutamyl-transpeptidase activity as well as bile acid uptake and export (Sampaziotis et al., 2015, 2017). Additionally, the use of laminin as a matrix increased the number and diameter of cysts as well as the expression of iPSC-derived cholangiocyte marker genes (Takayama et al., 2016).

### Adult Stem Cell-Derived 3D Liver Organoid Models

The liver displays a remarkable regenerative capacity (Michalopoulos, 2010; Hindley et al., 2014; Stanger, 2015). However, the types of cells involved in liver homeostasis and regeneration after damage are not well-defined. Previously, it was shown that Sox9-positive cells (oval cells) near the bile duct participate in the regenerative response of the liver after massive injury (Furuyama et al., 2011). Such findings are in close agreement with other studies that highlighted the contribution of biliary epithelial cells to the regenerative response of the liver (Dorrell et al., 2011; Espanol-Suner et al., 2012). Likewise, the results of these studies are consistent with other findings demonstrating that ablation of hepatic progenitors impairs liver regeneration (Shin et al., 2015). Collectively, these findings indicate that liver stem/progenitor cells retain the potential to form the liver parenchyma *in vivo* and organoids *in vitro*.

The available evidence suggests that Lgr5-positive progenitor cells are not present in the healthy adult liver and pancreas (Huch et al., 2013b), but, rather, they are activated upon chemical damage and become detectable near the bile ducts of the liver. It has been shown that freshly isolated healthy bile duct fragments and, more intriguingly, Lgr5-positive single cells isolated from mouse liver could yield 3D organoids. These bipotent epithelial liver organoids could be differentiated into mature and functional hepatocytes *in vitro* and could rescue liver failure in the fumarylacetoacetate hydrolase (*Fah*−/−) mutant mouse, a model for tyrosinemia type I liver disease, after transplantation (Huch et al., 2013b; Broutier et al., 2016). Furthermore, the Clevers group adapted a mouse liver organoid protocol to culture bipotent stem cells from the adult human liver (Huch et al., 2015). They demonstrated that human EpCAM-positive single cells from the ductal area or even ductal fragments could develop into clonal epithelial hepatic organoids. These organoids could generate functional hepatocytes after differentiation *in vitro* and engraftment within the parenchyma of recipient mouse liver *in vivo*. The study also found that the organoids were genetically stable in long-term cultures, being characterized by a low number of single base changes, most of which were probably incorporated before or during organoid generation but not during culture (Huch et al., 2015). Although Huch’s model elegantly demonstrated that the ductal progenitors had the capacity to generate hepatocytes under defined culture conditions and Raven and collaborators have more recently shown that impaired hepatocellular regeneration during liver injury could trigger ductular reaction and the generation of hepatocytes of non-hepatocyte origin (Raven et al., 2017), there is an ongoing controversy surrounding studies that demonstrated that the majority of regenerative response following hepatic damage rely primarily on hepatocytes rather than stem cells (Grompe, 2014). In particular, murine lineage-tracing studies provided supporting evidence that only hepatocytes were involved in liver regeneration after damage (Schaub et al., 2014; Yanger et al., 2014). Wnt signaling from liver endothelial cells can influence homeostatic self-renewal of hepatocytes. Specialized Axin2-positive cells localized to the pericentral liver lobule were previously identified as the source of precursors for this homeostatic reaction (Wang et al., 2015). The expression of early liver progenitor marker TBX3 in these cell types confirmed the presence of a mature and progenitor cell population in the adult liver. Likewise, periportal and hybrid hepatocytes with low levels of SOX9 expression replenished the liver mass after chronic injury (Font-Burgada et al., 2015). Accordingly, the Clevers group has recently established a new organoid model originating from a single mature Axin2-positive hepatocyte. Their study showed that Wnt/Rspo1 and HGF signaling pathways were the main regulators of primary hepatocyte expansion in both mouse and human organoid models. This approach facilitated the growth of hepatic organoids from a single hepatocyte directly after collagenase digestion. Here, both undamaged hepatocytes and hepatocytes from physically damaged liver (partial-hepatectomy, PHx) were used for the production of murine hepatic organoids. These organoids displayed the key functional attributes and gene expression profiles of hepatocytes. In addition, Gene Set Enrichment Analysis (GSEA) of PHx-derived organoids revealed that the gene expression profiles of mouse hepatic organoids resembled those of post-PHx proliferating hepatocytes. Accordingly, hepatic organoids recapitulated the regenerative response of adult liver upon partial hepatectomy. Fetal and adult human liver specimens were also used to form organoids in the same study. When compared to adult donors’ hepatic organoids, fetal-derived hepatic organoids displayed higher expansion capacity. One reason for impaired expansion in the adult hepatic organoids could be the inherently limited activity of telomerase enzyme (Hu et al., 2018). Moreover, the low number of cholangiocytes inside the hepatic organoids and trans-differentiation of Hep-org to Chol-org when cultured in cholangiocyte medium (Huch et al., 2015) is a sign of *de novo* generation of bile ducts after partial resection. Hepatic organoids, in this study, were different from the earlier Chol-org models in terms of cell size, nucleus/cytoplasm ratio, subcellular structure and function, and gene expression profiles. This critical difference could be explained by the cell of origin of the organoids. While the hepatic organoids originated from hepatocytes, Chol-org were developed from EpCAM-positive precursor cells. Moreover, in terms of *in vivo* functionality, the same study showed that organoids were able to engraft and repopulate in the liver of immunodeficient mice (Hu et al., 2018).

**FIGURE 2:**
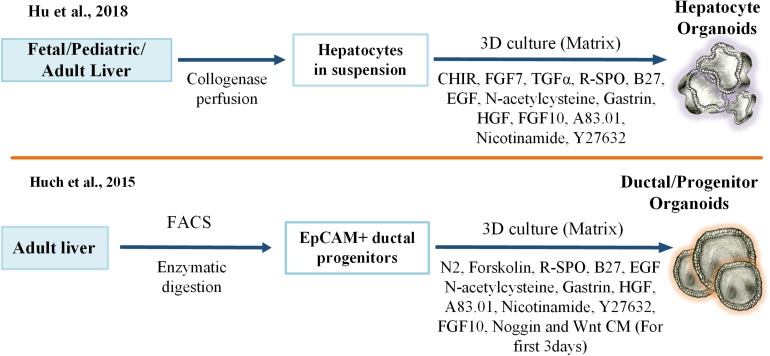
Key protocols for developing adult liver stem cell-derived 3D liver organoid cultures. 3D, three-dimensional, FGF, Fibroblast Growth Factor; TGF, Transforming Growth Factor; R-SPO, R-Spondin; EGF, Epidermal Growth Factor; HGF, Hepatocyte Growth Factor; CM, Condition Medium; FACS, Fluorescence-Activated Cell Sorting; EpCAM, Epithelial Cell Adhesion Molecule.

Complex crosstalk between hepatocytes, plasma, and other resident cells in the liver plays a key role in maintaining liver functions and regulating regenerative responses. Autocrine signals from hepatocytes (VEGF, TGF-α), paracrine signals from stellate cells (HGF) (Michalopoulos, 2007), and inflammatory cytokines from Kupffer cells (IL-6 and TNF-α) are essential for triggering a successful liver regenerative reaction. Peng et al. (2018) have recently shown that TNF-α would promote mouse hepatocyte expansion in 3D culture conditions *in vitro*. Also, these cells were able to engraft and repopulate in *Fah*−/− mouse liver. Based on these findings, one can conclude that this approach could potentially be integrated with Hu’s protocol for the generation of human hepatocytes with near-physiological features. Another liver organoid culture model has been developed by Vyas et al. (2017). Herein, decellularized ferret liver scaffolds were seeded with human fetal progenitor cells containing liver stromal and endothelial cells and then incubated in differentiation media for 3 weeks to induce hepatobiliary organoids in an *in vitro* culture setting. Although this model proved that hepatic and biliary lineage specification and maturation in scaffold was better than for the cells grown in Matrigel, the self-renewal capacity of the organoids and efficiency of this method in recapitulating adult liver functions was comparably low (Figure 2).

### 3D Organoid Models for Non-parenchymal Liver Cells

Hepatic stellate cells (HSCs) are liver-specific mesenchymal cells that contribute to liver physiology and pathophysiology (Scholten et al., 2010; Mederacke et al., 2013; Wells, 2014). In a healthy liver, HSCs are quiescent at baseline, and their function is to store vitamin A lipid droplets. Following damage due to toxins or viral infections, HSCs become metabolically active resulting in the accumulation of ECM in liver (Friedman, 2008). HSCs have limited proliferation capacity in 2D culture, cannot maintain the quiescent phenotype, and spontaneously lose key functional features *in vitro* (Mederacke et al., 2015; Perea et al., 2015). Koui et al. (2017) showed that iPSC-derived HSC progenitors could be expanded and maturated *in vitro* and could acquire lineage-specific characteristics. A recent study by Coll et al. (2018) has established an efficient culture system to differentiate HSCs from iPSCs. In this system, HSCs were generated via protocols that were initially developed to establish HSCs from ESCs (Asahina et al., 2009; Onitsuka et al., 2010). Briefly, iPSCs were differentiated toward mesodermal progenitors with BMP4. Then, these mesothelial cells were induced with retinol and palmitic acid for their subsequent differentiation into HSCs. After 12 days, the resulting cells resembled primary HSCs in terms of morphology, transcriptome profile, and functional attributes. In addition, when grown as 3D spheroid co-cultures together with HepaRG cells, HSCs stored vitamin A and, more importantly, they switched from a quiescent state to an activated state in response to hepatic toxicity. In conclusion, this method provided a robust and reliable culture system for the investigation of liver biology and pathobiology using hepatic organoids and activated HSCs (Coll et al., 2018).

## Potential Applications of Liver Organoids

**FIGURE 3:**
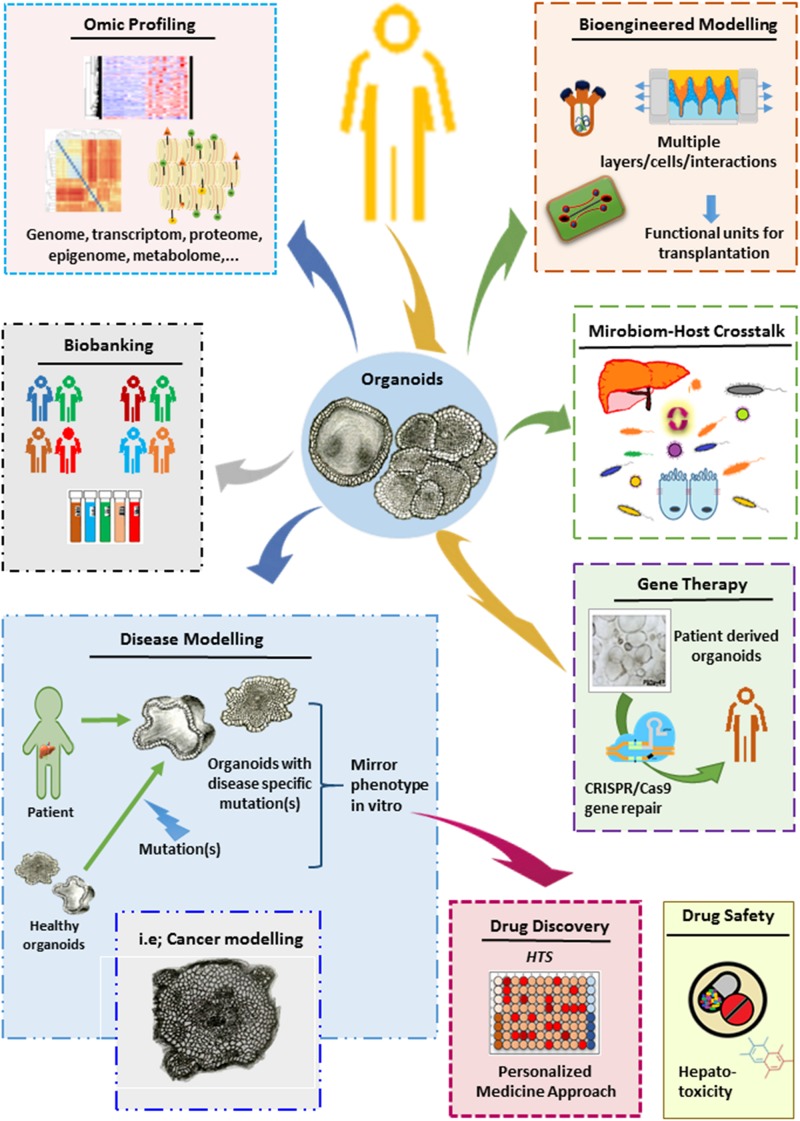
Potential applications for liver organoids.

Organoids have many great advantages over conventional cell culture techniques. These miniaturized organs enable long-term stable expansion in a near-physiological manner and mimic 3D tissue function and structure and disease pathology. Therefore, they have tremendous potential to be transformed into excellent platforms for various basic and translational research applications, including disease modeling, drug screening, gene therapy, elucidating microbe-host interactions, and organ replacement (Figure 3).

### Disease Models via Liver Organoids

Over the past few years, with the discovery of other powerful technologies, CRISPR-Cas9 genome editing in particular, organoids research has opened up new avenues for understanding the molecular pathophysiology of diseases. To this end, the introduction of disease-specific mutations into the liver organoids derived from healthy donors has become rapid and seamless, enabling researchers to readily investigate mutation-related mechanisms and clinical phenotypes. Alternatively, tissue explants or biopsies from patients can also be used directly for the production of disease-specific organoids. Table 1 summarizes the liver disease models published in the literature.

**TABLE 1:** Organoid-based liver disease models.

**Organoid type**	**Cell source**	**Disease**	**Modeling approach**		**References**
			**Patient-derived**	**Genome editing**	
Hepatic	Liver Biopsy	A1-Antitrypsin Deficiency, Alagille Syndrome	✓		Huch et al., 2015
Tumor	Surgical Resection	Liver Cancer	✓		Broutier et al., 2017
Tumor	Needle Biopsy	Liver Cancer	✓		Nuciforo et al., 2018
Tumor	Surgical Resection	Liver Cancer	✓		Li et al., 2019
Hepatic	iPSCs	Alagille Syndrome	✓	✓	Guan et al., 2017
Hepatic	iPSCs	Citrullinemia type I	✓		Akbari et al., 2019
Hepatic	iPSCs	Steatohepatitis Wolman Disease	✓		Ouchi et al., 2019
Cholangiocyte	iPSCs	Cystic Fibrosis	✓		Ogawa et al., 2015
Cholangiocyte	iPSCs	Cystic Fibrosis	✓		Sampaziotis et al., 2015

#### Rare and Genetic Diseases

Alpha-1-antitrypsin deficiency (A1ATD) is a rare inherited disorder that causes lung and liver diseases, skin problems, and inflammation of the blood vessels. A1AT is a serine protease inhibitor that is synthesized mostly in the liver and transferred through the bloodstream to the lungs. Its primary function in the lungs is to inhibit an enzyme called neutrophil elastase, thus protecting the lung tissue against proteolytic degradation. The most common genetic missense mutation in A1ATD is the PiZ allele (Glu^342^Lys) of the SERPINA1 gene, which results in the agglomeration of misfolded proteins in hepatocytes (Bals, 2010). To model this condition, Huch et al. (2015) developed organoids using liver biopsy specimens obtained from three patients with A1ATD disease. Notably, patient-derived organoids (PDO) mimicked the disease pathology, with reduced protein levels and aggregation of A1AT protein in the endoplasmic reticulum. Alagille syndrome (ALGS), another multisystem genetic disorder, was also modeled using the same approach (Huch et al., 2015). Of note, ALGS has autosomal dominant inheritance and is predominantly caused by heterozygous mutations in the JAG1 gene, which codes for the Jagged1 protein, a transmembrane ligand in the Notch signaling pathway. JAG1 mutations lead to deficiencies in various tissues, including liver, heart, and muscle (Turnpenny and Ellard, 2012). According to the findings of this study, organoids from ALGS patients were unable to form intact bile duct structures *in vitro*, and cells in the organoid lumen underwent apoptosis, mimicking the *in vivo* disease pathology (Huch et al., 2015). In addition to liver stem/progenitor-derived organoids, Guan and colleagues used patient-specific iPSCs to model this genetic disorder. Specifically, iPSCs from two ALGS patients were differentiated into organoids via the protocol described in Figure 1. In contrast to healthy organoids, the bile duct formation and the biliary transport function of ALGS-specific PDOs were significantly impaired. In addition, using a reverse approach employing CRISPR-Cas9 technology, ALGS-causing JAG1 mutation C829X was introduced into iPSCs prepared from healthy human fibroblasts. As a result, the capacity of the genetically modified iPSC-derived organoids to form duct-like structures was significantly reduced, recapitulating the ALGS liver pathology (Guan et al., 2017).

Citrullinemia type I (CTLN1) is an inherited urea cycle disorder of the liver arising from a deficiency in the enzyme Argininosuccinate Synthase 1 (ASS1) (Kose et al., 2017). Infant patients with severe Citrullinemia type I experience life-threatening clinical manifestations, such as episodes of hyperammonemia that might be fatal or result in permanent neurologic damage. Current strategies (drugs and a low-protein diet) for the management of Citrullinemia type I are not effective, and, in severe cases, long-term survival is low. Cell transplantation with functional hepatocytes emerges as a promising therapeutic approach. Recently, iPSCs derived from Citrullinemia type I patients with homozygous G390R mutations in the ASS1 gene have been used to develop a disease-specific model. Particularly, patient-specific iPSCs were generated by reprograming dermal fibroblasts with episomal vectors and differentiating toward definitive endoderm under defined conditions. Then, iPSC-derived EpCAM-positive endodermal cells were isolated and used to generate functional hepatic organoid cultures. These organoids exhibited hepatocyte-specific marker gene expression and recapitulated the key metabolic functions of mature hepatocytes, including LDL uptake, albumin secretion, and glycogen storage. Further analysis revealed that the hepatic organoids mimicked the clinical phenotypes of Citrullinemia type I patients such as increased ammonia and decreased urea. Moreover, these phenotypes could be rescued by ectopic expression of the wild-type ASS1 gene in patient-derived organoids. Collectively, this model offers a promising framework to study gene correction/replacement strategies in cell-based therapies and perform pre-clinical drug discovery and development studies in urea cycle disorders (Akbari et al., 2019).

Cholangiocyte organoids derived from iPSCs have also been used for disease modeling. For instance, Ogawa and colleagues, as described in Figure 1, used patient-specific Chol-org for modeling the Cystic Fibrosis (CF) disease. CF is a life-threatening rare autosomal recessive disorder caused by mutations in the Cystic Fibrosis Transmembrane Conductance Regulator (CFTR) gene. Mutations in the CFTR gene disrupt the function of the chloride ion channel, causing dysregulation of ion flux and epithelial fluid transport in the lung, pancreas, colon, and liver. In this study, Chol-org derived from iPSCs displayed misfolded CFTR proteins, impaired cyst formation, and loss of chloride channel function, reflecting the disease phenotype of the patients. Furthermore, they showed that exposure of the organoids to a small-molecule CFTR potentiator drug VX-770 (Ivacaftor) along with forskolin and IBMX corrected the misfolding defect and led to the functional restoration of the mutant CFTR protein (Ogawa et al., 2015). Chol-org were also used in another study to successfully model ALGS, polycystic liver disease, and CF-associated cholangiopathy (Sampaziotis et al., 2015). Particularly, the treatment of patient-derived Chol-org with CF-related drugs (verapamil and octreotide) rescued the clinical phenotype of CF cholangiopathy *in vitro*. In support of these findings, an ongoing research project called HIT-CF Europe^1^ is also using next-generation cell culture systems with the aim of evaluating the efficacy and safety of rare CF mutation-specific drug candidates in patient-derived organoids. The project highlights the exciting promise of organoid culture systems to provide better lives for people with CF. Taken together, next-generation organoid technologies hold great potential to accurately model biliary-related diseases and pave the way for organoid-based preclinical studies in precision medicine.

#### Cancer and Complex Diseases

Liver cancer is the fifth most common cancer in the world and the second leading cause of death (Bray et al., 2018). The lack of powerful liver cancer models that accurately recapitulate the histopathology, heterogeneity, genome and transcriptome profile of tumors hampers the development of effective treatments. While different liver cancer models, such as cancer cell lines and patient-derived xenografts (PDX), have been established in the past, the drawbacks of each model limit their applications in liver cancer studies. For instance, when passaged for long periods in 2D culture, the cancer cell lines accumulate mutations and undergo clonal selection, which results in loss of original genetic and phenotypic heterogeneity (Kim, 2005). Moreover, the low engraftment rate, high cost, time-consuming nature of establishment, and non-suitability for large-scale drug testing limit the use of PDX models (Hidalgo et al., 2014; Gu et al., 2015; Cavalloni et al., 2016).

Personalized cancer medicine is a novel and innovative approach aiming to establish effective therapeutic strategies for each patient based on tumor-specific features. In this regard, tumor organoids called tumoroid models are emerging as a promising platform. Recently, Broutier et al. established a tumoroid culture model from three different subtypes of primary liver cancer (PLC), namely hepatocellular carcinoma (HCC), cholangiocarcinoma (CC), and combined hepatocellular-cholangiocarcinoma (CHC), using liver cancer samples obtained from patients with no history of viral treatment. The established tumoroids retained the histological characteristics as well as transcriptome profiles and genomic signatures of the original tissue. Furthermore, *in vivo* studies demonstrated the tumorigenic and metastatic potential of PLC-derived organoids upon transplantation under the kidney capsule. Importantly, integrated omics data from PLC-derived organoids led the researchers to identify new biomarkers and novel potential therapeutic agents for PLC, such as an ERK inhibitor (Broutier et al., 2017). In another study, Nuciforo et al. (2018) developed a methodology through which they generated tumoroids from needle biopsy samples from different HCC patients with various tumor stages and etiologies. These tumoroids successfully recapitulated the histopathological properties along with the gene expression profiles and mutational landscapes of the original tissues. Moreover, they also preserved tumor features after transplantation into immunodeficient mice (Nuciforo et al., 2018).

Recently, Li et al. (2019) have developed a cohort of 27 tumoroid cell lines from multiple regions of human tumor samples from CC and HCC patients. Epithelial, bile duct, stemness, and mucin markers were used for the characterization of the cellular identity of tumoroids. Further analysis demonstrated that the patient-derived tumoroids reflected the histological features, transcriptome profiles and genetic background of the original primary liver tumors. Interestingly, high-throughput drug screening of these tumoroids displayed heterogeneous drug responses, suggesting strong intra- and inter-tumor heterogeneity. As a result, only a small group of drugs was found to be effective in blocking their proliferation (Li et al., 2019).

With regard to liver cancer research, a new study demonstrated that organoid technology could be utilized to model cancer initiation (Sun et al., 2019). Specifically, Sun and colleagues suggested that direct reprograming of fibroblasts into 3D hepatocytes could be accomplished by ectopic expression of FOXA3, HNF1A, and HNF4A. Intriguingly, these hepatic organoids had limited expansion and proliferation capacity, which could be overcome by SV40 transduction. In this model, c-MYC transduction facilitated HCC initiation and liver cancer formation *in vivo*, in part through the induction of excessive mitochondrion-endoplasmic reticulum coupling. Furthermore, when transplanted orthotopically, RAS-transformed organoids formed intra-hepatic cholangiocarcinomas with a hepatocyte origin (Sun et al., 2019).

Organoids derived from normal tissues can also be used to model cancer by sequential introduction of cancer driver gene mutations. The advantage of using such models is they enable investigation of the effects of driver mutations in an isogenic (identical) genetic background. To this end, different laboratories have independently engineered mutant colon organoids by incorporating mutations for at least four of the most commonly altered genes in colorectal carcinoma, namely KRAS, APC, TP53, SMAD4, and PIK3CA (Drost et al., 2015; Matano et al., 2015). These mutant organoids developed adenocarcinomas when transplanted into the kidney capsule of recipient mice, modeling the progression of colon cancer. Similarly, a recent study has revealed that BAP1 loss-of-function by CRISPR-Cas9 in liver organoids, in combination with common cholangiocarcinoma mutations (TP53, PTEN, SMAD4, and NF1), affects epithelial tissue organization and cell-to-cell junctions and results in the acquisition of malignant features (Artegiani et al., 2019).

The potential of human liver organoids as a model for studying chronic and complex liver diseases is also promising for the discovery of effective treatments. In that regard, Ouchi et al. (2019) have developed a method to generate a multi-cellular liver organoid system in order to model non-alcoholic steatohepatitis. In particular, the liver organoids exhibited lipid accumulation, fibrosis, and inflammation upon free fatty acid exposure. The findings of this study suggest that this system could recapitulate the progressive aspects of steatohepatitis and model Wolman disease and, more importantly, that FGF19 could be used to alleviate the disease pathologies (Ouchi et al., 2019).

### Host–Microbiome Interactions

Randall’s laboratory has studied Hepatitis C Virus (HCV) entry and localization in hepatoma organoids. HCV colocalizes in basolateral membrane, and its virions progressively accumulate at tight junctions. This model is a promising tool for investigating the complex traffic processes of HCV (Baktash et al., 2018). Recently, Taniguchi’s laboratory generated functional human iPSC-derived liver organoids and infected them with the Hepatitis B Virus (HBV) genome. Herein, the functional liver organoids demonstrated higher susceptibility to HBV infection than human iPSC-derived 2D hepatic-like cells and could maintain HBV propagation and produce infectious virus for longer durations. Notably, HBV infection resulted in severe hepatic dysfunction of organoids, characterized by elevated hepatic injury markers and an altered hepatic ultrastructure. Interestingly, this study also showed that HBV-infected liver organoids generated without the immune cells had an impaired hepatic function and thus proposed the hypothesis that HBV might indeed be a cytopathic virus (Nie et al., 2018).

In summary, liver organoids are highly promising models for studying hepatitis virus-host interactions and can easily become personalized infection models for individualized hepatitis studies and treatments.

### Omic Profiling

Instead of screening from established drug libraries, omics data can be used to predict novel drug candidates for diseases. With the help of organoid technology, one could also amplify sufficient quantities of healthy and diseased tissues *in vitro* and analyze causal mutations by deep sequencing or track treatment regimens by phenotypic profiling of the organoids using omic platforms. Recently, Huch’s team has predicted novel drug targets for liver cancer patients by studying the omic profiles of tumoroids and showed that ERK inhibitors could decrease tumor formation in patient-derived xenograft models (Broutier et al., 2017). These findings indicate that omic profiling would offer a convenient strategy to understand the molecular pathogenesis of diseases and identify novel therapeutic drug targets.

### Bioengineered Organoid Models

To increase the longevity and reproducibility of cell functions *in vitro*, the research community is now using multiple bioengineering tools such as high-throughput microarrays, lab-on-chip technologies (Polini et al., 2014; Bhise et al., 2016), protein micropatterning, microfluidics, specialized plates, biomimetic scaffolds, and bioprinting to control the cellular microenvironment (Zhang et al., 2017b). Underhill and Khetani (2018) have recently reviewed the advances in bioengineered liver models with utility in drug screening and the microenvironmental determinants of liver cell differentiation/function (Massa et al., 2017; Zhang et al., 2017a).

Jin et al. (2018) developed a vascularized 3D liver organoid model derived from induced hepatocytes (iHep) that were directly differentiated from fibroblasts. They achieved this by co-culturing the iHeps with endothelial cells in a decellularized 3D liver extracellular matrix (LEM) hydrogel in a microfluidic-based cell culture device with a continuous dynamic flow of media. By taking advantage of this platform, iHep-based 3D liver organoids were able to establish a multiorgan system comprised of multiple organoids derived from different internal organs and demonstrated great feasibility for functional and standardized high-throughput drug screening (HTS) (Jin et al., 2018).

Micro-scale technologies can mimic *in vivo*-like cellular microenvironments by allowing precise control of crucial physicochemical factors such as fluid flow, biochemical signals, and so on (Huh et al., 2010; Schepers et al., 2016; Chung et al., 2017). Recently, Wang et al. (2018) proposed a simple and robust strategy that promoted *in situ* differentiation, long-term 3D culture, and the generation of hiPSC-based functional liver organoids in a perfusable micropillar chip system. The liver organoids displayed favorable growth and cellular heterogeneity characteristics involving the differentiation of hepatocytes and cholangiocytes, mimicking liver tissue *in vivo*. In particular, the liver organoids under perfused culture conditions exhibited improved cell viability as well as endoderm- and mature hepatocyte-specific gene expression. Moreover, when compared to static cultures, they displayed enhanced metabolic capabilities and liver-specific functions including albumin and urea production. Finally, the liver organoids showed a dose- and time-dependent hepatotoxic response to acetaminophen, which again offers an ideal platform for drug testing (Wang et al., 2018).

### Hepatotoxicity

Hepatotoxicity refers to the toxic effects of drugs and their metabolites on liver tissue and usually implies chemical-driven acute liver injury and failure. Toxicity analysis for all kinds of drugs is essential prior to their approval for entry into the market (Kaplowitz, 2005). Up to now, 2D primary hepatocytes have been used as a model for drug metabolism and toxicity screening. However, hepatocytes are unstable and functionally ineffective in long-term cultures due to impaired cytochrome P450 (CYP) activity. To this end, hepatic organoids would offer an excellent platform on which to perform chemical-induced hepatotoxicity prediction. Recently, Mun et al. (2019) have found that hepatic organoids can express Phase I drug metabolism CYP enzymes and Phase II detoxification enzymes. Specifically, CYP1A2 and CYP3A4 were induced following omeprazole treatment in hepatic organoid culture. In addition, CYP3A4- and CYP1A2/2E1-mediated hepatotoxic drugs (troglitazone and APAP, respectively) were analyzed in 2D hepatocyte and organoid cultures. Based on cell viability experiments following exposure to hepatotoxic drugs at clinically relevant concentrations, hepatic organoids exhibited higher sensitivity than 2D hepatocytes. In particular, the toxic concentration of trovafloxacin (a broad-spectrum antibiotic) was approximately 50-fold higher in 2D hepatocytes (Mun et al., 2019). In conclusion, organoid-based hepatotoxicity analysis proves beneficial for drug screening studies and early prediction of chemical-induced liver injury. Alternatively, our recent findings suggest that iPSC-derived hepatic organoids can be readily cultured for many generations (at least 48 passages) and continue to preserve hepatic functions, rendering this model suitable for long-term preclinical hepatotoxicity screening studies (Akbari et al., 2019).

### Biobanking

The organoid models will also serve as a renewable resource via cryopreserved biobanks of healthy and diseased human organoids (van de Wetering et al., 2015). Today, these “Living Biobanks” are becoming increasingly attractive to researchers in academia and industry for various purposes, especially those related to the development of innovative therapeutic strategies, the identification of novel diagnostic markers, and the development of individualized patient treatment plans. Recently, the Human Cancer Models Initiative (HCMI), was established as an international collaboration between the US National Cancer Institute (NCI), Cancer Research UK (CRUK), the Wellcome Sanger Institute (WSI), and the Hubrecht Organoid Technology (The HUB). As part of the pilot phase, by using cutting-edge technologies, the HCMI aims to generate, clinically annotate, and genetically characterize around 1,000 next-generation cancer cell/organoid models from patient tumors^2^. The overall goal of the HCMI is to advance cancer research by providing the research community with a large collection of readily accessible cancer cell models and all the necessary resources, including consent forms and standardized protocols, used for model development. These cancer models can then be utilized to perform basic and translational cancer research and contribute to drug target discovery, the identification of novel disease-specific biomarkers, and the development of preventive as well as therapeutic strategies for personalized medicine.

### Gene Therapy

Targeted gene therapy has been used with great success to repair or inactivate mutations of genetic diseases in animal or *in vitro* cell culture models. In the near future, many efforts will likely be devoted to combining modern genome editing tools with organoid technologies *in vitro* to generate healthy isogenic organoids by repairing genetic defects in patient-derived organoids. Hopefully, these technologies could potentially be exploited to treat patients with life-threatening and otherwise incurable diseases, granted that the grafts are accepted by the recipient tissue without any immune rejection. As an example, a disease-causing mutation has recently been reverted to wild-type via CRISPR-Cas9 in patient-derived organoids from Alagille syndrome, and the phenotype of the disease was successfully rescued *in vitro* (Guan et al., 2017). We have reason to believe that whenever the genome editing technologies become fully applicable for clinical practice with regard to safety and efficiency, organoid technologies will also become feasible for such therapies.

### Transplantation

Functional organoids are promising alternatives for cell and whole organ transplantations, as they can be produced from isogenic self-tissues. Using iPSC technologies, it is also possible to generate HLA-matched tissue-specific organoids from readily accessible tissue biopsies. Indeed, the capacity of *in vitro* cultured organoids to repair diseased or damaged tissue *in vivo* has been demonstrated by studies reporting functional engraftment of orthotopically transplanted organoids in the kidney (van den Berg et al., 2018), liver (Takebe et al., 2013; Huch et al., 2015; Hu et al., 2018; Akbari et al., 2019), and brain (Mansour et al., 2018). As a final remark, these transplantable organoids should be produced following method validation, a key element in proving the quality and reliability of the product being developed, under Good Manufacturing Laboratory (GMP) compliance.

## Limitations

Even though the volume of literature on this topic has exploded incredibly over the course of the last few years (named Method of the Year 2017, 2018), the organoid technology remains an imperfect cell culture model, and various challenges and limitations need to be addressed to improve organoid models. Currently, the time required for tissue maturation and for organoid outgrowth is a major limitation, increasing the cost of organoid generation, particularly for certain organoids such as retina, brain, and liver, where early progenitor/undifferentiated cells are present in the structure. Other limitations of organoid culture are the lack of vascularization, which is essential for nutrient exchange, and the lack of interaction with other cell types of the native microenvironment, such as immune and neural cells. Moreover, current organoid cultures fail to recapitulate the complex network between different body systems, which is a limiting factor in studying the coordinated function and crosstalk between distinct organs. In addition, organoid cultures are heterogeneous, with no reliable means of synchronizing size, shape, and viability. This, unfortunately, leads to complications in systematic data analysis and study design (Fatehullah et al., 2016). Additionally, Matrigel is often a critical component of organoid culture. Matrigel derived from mouse-sarcoma is the major practical barrier against the generation of human-transplantable organoids. Furthermore, organoid culture is unable to mimic *in vivo* growth factor/signaling gradients in matrix, a potential limitation for organoid growth from certain tissues. To address these challenges and overcome these limitations, biomimetic scaffolds based on either synthetic polymers or natural macromolecules need to be constructed, and protocols regarding media recipes need to be modified.

## Discussion

Organoids are one of the most accessible 3D cultures of cells and organ fragments, in which the self-organizing properties of stem cells and their differentiated progeny are orchestrated to establish physiologically relevant models of human tissues *in vitro*. These rapidly evolving models have a wide range of applications, such as providing a source of functional healthy and diseased human tissue from limited amounts of starting material for studying tissue-specific biological processes, analyzing the dynamics of stem cell behavior, and performing drug screening and disease modeling at near-physiological conditions, thus maximizing their potential to bridge the gap between basic research and translational medicine. Increasing interest in organoid technologies will ensure that these models are accessible to a broad range of academic and clinical scientists. In combination with a more defined ECM, it can be foreseen that a highly accurate and reproducible culture model could emerge and overcome existing constraints that prevent the transition from bench to bedside.

Over the past decade, liver organoids have proved the most powerful next-generation cell culture system in modeling liver diseases and are becoming an increasingly viable option for disease- or patient-specific therapeutic strategies in personalized liver medicine.

## Author Contributions

All of the authors researched the literature for the manuscript, made substantial contributions to the discussion of the content, and wrote the manuscript. SA, SS, and EE reviewed and edited the manuscript.

## Conflict of Interest

The authors declare that the research was conducted in the absence of any commercial or financial relationships that could be construed as a potential conflict of interest.

## Footnotes

1 https://www.hitcf.org/

2 https://ocg.cancer.gov/programs/hcmi

## References

[B1] Aizarani N. Saviano A. Sagar, Mailly L. Durand S. Herman J. S. (2019). A human liver cell atlas reveals heterogeneity and epithelial progenitors. *Nature* 572 199–204. 10.1038/s41586-019-1373-2 31292543 PMC6687507

[B2] Akbari S. Sevinç G. G. Ersoy N. Basak O. Kaplan K. Sevinç K. (2019). Robust, long-term culture of endoderm-derived hepatic organoids for disease modeling. *Stem Cell Rep.* 13 627–641. 10.1016/j.stemcr.2019.08.007 31522975 PMC6829764

[B3] Artegiani B. Van Voorthuijsen L. Lindeboom R. G. H. Seinstra D. Heo I. Tapia P. (2019). Probing the tumor suppressor function of BAP1 in CRISPR-engineered human liver organoids. *Cell Stem Cell* 24 927–943.e6. 10.1016/j.stem.2019.04.017 31130514

[B4] Asahina K. Tsai S. Y. Li P. Ishii M. Maxson R. E. Sucov H. M. (2009). Mesenchymal origin of hepatic stellate cells, submesothelial cells, and perivascular mesenchymal cells during mouse liver development. *Hepatology* 49 998–1011. 10.1002/hep.22721 19085956 PMC2673117

[B5] Asrani S. K. Devarbhavi H. Eaton J. Kamath P. S. (2019). Burden of liver diseases in the world. *J. Hepatol.* 70 151–171. 10.1016/j.jhep.2018.09.014 30266282

[B6] Baktash Y. Madhav A. Coller K. E. Randall G. (2018). Single particle imaging of polarized hepatoma organoids upon hepatitis C virus infection reveals an ordered and sequential entry process. *Cell Host Microbe* 23 382–394.e5. 10.1016/j.chom.2018.02.005 29544098 PMC7169308

[B7] Bals R. (2010). Alpha-1-antitrypsin deficiency. *Best Pract. Res. Clin. Gastroenterol.* 24 629–633. 10.1016/j.bpg.2010.08.006 20955965

[B8] Barker N. Huch M. Kujala P. Van De Wetering M. Snippert H. J. Van Es J. H. (2010). Lgr5(+ve) stem cells drive self-renewal in the stomach and build long-lived gastric units in vitro. *Cell Stem Cell* 6 25–36. 10.1016/j.stem.2009.11.013 20085740

[B9] Baxter M. Withey S. Harrison S. Segeritz C. P. Zhang F. Atkinson-Dell R. (2015). Phenotypic and functional analyses show stem cell-derived hepatocyte-like cells better mimic fetal rather than adult hepatocytes. *J. Hepatol.* 62 581–589. 10.1016/j.jhep.2014.10.016 25457200 PMC4334496

[B10] Bhise N. S. Manoharan V. Massa S. Tamayol A. Ghaderi M. Miscuglio M. (2016). A liver-on-a-chip platform with bioprinted hepatic spheroids. *Biofabrication* 8:014101. 10.1088/1758-5090/8/1/014101 26756674

[B11] Bissell D. M. Arenson D. M. Maher J. J. Roll F. J. (1987). Support of cultured hepatocytes by a laminin-rich gel. Evidence for a functionally significant subendothelial matrix in normal rat liver. *J. Clin. Invest.* 79 801–812. 10.1172/JCI112887 3546380 PMC424203

[B12] Boj S. F. Hwang C. I. Baker L. A. Chio I. I. Engle D. D. Corbo V. (2015). Organoid models of human and mouse ductal pancreatic cancer. *Cell* 160 324–338. 10.1016/j.cell.2014.12.021 25557080 PMC4334572

[B13] Boonekamp K. E. Kretzschmar K. Wiener D. J. Asra P. Derakhshan S. Puschhof J. (2019). Long-term expansion and differentiation of adult murine epidermal stem cells in 3D organoid cultures. *Proc. Natl. Acad. Sci. U.S.A.* 116 14630–14638. 10.1073/pnas.1715272116 31253707 PMC6642409

[B14] Boost K. A. Auth M. K. Woitaschek D. Kim H. S. Hilgard P. Nadalin S. (2007). Long-term production of major coagulation factors and inhibitors by primary human hepatocytes in vitro: perspectives for clinical application. *Liver Int.* 27 832–844. 10.1111/j.1478-3231.2007.01472.x 17617127

[B15] Bray F. Ferlay J. Soerjomataram I. Siegel R. L. Torre L. A. Jemal A. (2018). Global cancer statistics 2018: GLOBOCAN estimates of incidence and mortality worldwide for 36 cancers in 185 countries. *CA Cancer J. Clin.* 68 394–424. 10.3322/caac.21492 30207593

[B16] Broutier L. Andersson-Rolf A. Hindley C. J. Boj S. F. Clevers H. Koo B. K. (2016). Culture and establishment of self-renewing human and mouse adult liver and pancreas 3D organoids and their genetic manipulation. *Nat. Protoc.* 11 1724–1743. 10.1038/nprot.2016.097 27560176

[B17] Broutier L. Mastrogiovanni G. Verstegen M. M. Francies H. E. Gavarro L. M. Bradshaw C. R. (2017). Human primary liver cancer-derived organoid cultures for disease modeling and drug screening. *Nat. Med.* 23 1424–1435. 10.1038/nm.4438 29131160 PMC5722201

[B18] Camp J. G. Sekine K. Gerber T. Loeffler-Wirth H. Binder H. Gac M. (2017). Multilineage communication regulates human liver bud development from pluripotency. *Nature* 546 533–538. 10.1038/nature22796 28614297

[B19] Cavalloni G. Peraldo-Neia C. Varamo C. Casorzo L. Dell’aglio C. Bernabei P. (2016). Establishment and characterization of a human intrahepatic cholangiocarcinoma cell line derived from an Italian patient. *Tumour Biol.* 37 4041–4052. 10.1007/s13277-015-4215-3 26486326 PMC4844644

[B20] Chen Y. F. Tseng C. Y. Wang H. W. Kuo H. C. Yang V. W. Lee O. K. (2012). Rapid generation of mature hepatocyte-like cells from human induced pluripotent stem cells by an efficient three-step protocol. *Hepatology* 55 1193–1203. 10.1002/hep.24790 22095466 PMC3779307

[B21] Chung M. Ahn J. Son K. Kim S. Jeon N. L. (2017). Biomimetic model of tumor microenvironment on microfluidic platform. *Adv. Healthc. Mater.* 6:1700196. 10.1002/adhm.201700196 28544639

[B22] Clayton D. F. Darnell J. E. Jr. (1983). Changes in liver-specific compared to common gene transcription during primary culture of mouse hepatocytes. *Mol. Cell. Biol.* 3 1552–1561. 10.1128/mcb.3.9.1552 6633533 PMC370008

[B23] Clevers H. (2016). Modeling development and disease with organoids. *Cell* 165 1586–1597. 10.1016/j.cell.2016.05.082 27315476

[B24] Coll M. Perea L. Boon R. Leite S. B. Vallverdu J. Mannaerts I. (2018). Generation of hepatic stellate cells from human pluripotent stem cells enables in vitro modeling of liver fibrosis. *Cell Stem Cell* 23 101–113.e7. 10.1016/j.stem.2018.05.027 30049452

[B25] D’Amour K. A. Agulnick A. D. Eliazer S. Kelly O. G. Kroon E. Baetge E. E. (2005). Efficient differentiation of human embryonic stem cells to definitive endoderm. *Nat. Biotechnol.* 23 1534–1541. 10.1038/nbt1163 16258519

[B26] Darnell M. Ulvestad M. Ellis E. Weidolf L. Andersson T. B. (2012). In vitro evaluation of major in vivo drug metabolic pathways using primary human hepatocytes and HepaRG cells in suspension and a dynamic three-dimensional bioreactor system. *J. Pharmacol. Exp. Ther.* 343 134–144. 10.1124/jpet.112.195834 22776955

[B27] Dianat N. Dubois-Pot-Schneider H. Steichen C. Desterke C. Leclerc P. Raveux A. (2014). Generation of functional cholangiocyte-like cells from human pluripotent stem cells and HepaRG cells. *Hepatology* 60 700–714. 10.1002/hep.27165 24715669 PMC4315871

[B28] Dorrell C. Erker L. Schug J. Kopp J. L. Canaday P. S. Fox A. J. (2011). Prospective isolation of a bipotential clonogenic liver progenitor cell in adult mice. *Genes Dev.* 25 1193–1203. 10.1101/gad.2029411 21632826 PMC3110957

[B29] Douarin N. M. (1975). An experimental analysis of liver development. *Med. Biol.* 53 427–455. 765644

[B30] Drost J. Karthaus W. R. Gao D. Driehuis E. Sawyers C. L. Chen Y. (2016). Organoid culture systems for prostate epithelial and cancer tissue. *Nat. Protoc.* 11 347–358. 10.1038/nprot.2016.006 26797458 PMC4793718

[B31] Drost J. Van Jaarsveld R. H. Ponsioen B. Zimberlin C. Van Boxtel R. Buijs A. (2015). Sequential cancer mutations in cultured human intestinal stem cells. *Nature* 521 43–47. 10.1038/nature14415 25924068

[B32] Duval K. Grover H. Han L. H. Mou Y. Pegoraro A. F. Fredberg J. (2017). Modeling physiological events in 2D vs. 3D cell culture. *Physiology* 32 266–277. 10.1152/physiol.00036.2016 28615311 PMC5545611

[B33] Dye B. R. Hill D. R. Ferguson M. A. H. Tsai Y.-H. Nagy M. S. Dyal R. (2015). In vitro generation of human pluripotent stem cell derived lung organoids. *eLife* 4:e05098. 10.7554/eLife.05098 25803487 PMC4370217

[B34] Eiraku M. Takata N. Ishibashi H. Kawada M. Sakakura E. Okuda S. (2011). Self-organizing optic-cup morphogenesis in three-dimensional culture. *Nature* 472 51–56. 10.1038/nature09941 21475194

[B35] Eldred K. C. Hadyniak S. E. Hussey K. A. Brenerman B. Zhang P.-W. Chamling X. (2018). Thyroid hormone signaling specifies cone subtypes in human retinal organoids. *Science* 362:eaau6348. 10.1126/science.aau6348 30309916 PMC6249681

[B36] Espanol-Suner R. Carpentier R. Van Hul N. Legry V. Achouri Y. Cordi S. (2012). Liver progenitor cells yield functional hepatocytes in response to chronic liver injury in mice. *Gastroenterology* 143 1564–1575.e7. 10.1053/j.gastro.2012.08.024 22922013

[B37] Fatehullah A. Tan S. H. Barker N. (2016). Organoids as an in vitro model of human development and disease. *Nat. Cell Biol.* 18 246–254. 10.1038/ncb3312 26911908

[B38] Fiorotto R. Amenduni M. Mariotti V. Fabris L. Spirli C. Strazzabosco M. (2018). Liver diseases in the dish: iPSC and organoids as a new approach to modeling liver diseases. *Biochim. Biophys. Acta Mol. Basis Dis.* 1865 920–928. 10.1016/j.bbadis.2018.08.038 30264693 PMC6658095

[B39] Font-Burgada J. Shalapour S. Ramaswamy S. Hsueh B. Rossell D. Umemura A. (2015). Hybrid periportal hepatocytes regenerate the injured liver without giving rise to cancer. *Cell* 162 766–779. 10.1016/j.cell.2015.07.026 26276631 PMC4545590

[B40] Friedman S. L. (2008). Hepatic stellate cells: protean, multifunctional, and enigmatic cells of the liver. *Physiol. Rev.* 88 125–172. 10.1152/physrev.00013.2007 18195085 PMC2888531

[B41] Furuyama K. Kawaguchi Y. Akiyama H. Horiguchi M. Kodama S. Kuhara T. (2011). Continuous cell supply from a Sox9-expressing progenitor zone in adult liver, exocrine pancreas and intestine. *Nat. Genet.* 43 34–41. 10.1038/ng.722 21113154

[B42] Gordillo M. Evans T. Gouon-Evans V. (2015). Orchestrating liver development. *Development* 142 2094–2108. 10.1242/dev.114215 26081571 PMC4483763

[B43] Greggio C. De Franceschi F. Figueiredo-Larsen M. Gobaa S. Ranga A. Semb H. (2013). Artificial three-dimensional niches deconstruct pancreas development in vitro. *Development* 140 4452–4462. 10.1242/dev.096628 24130330 PMC4007719

[B44] Grompe M. (2014). Liver stem cells, where art thou? *Cell Stem Cell* 15 257–258. 10.1016/j.stem.2014.08.004 25192457

[B45] Gu Q. Zhang B. Sun H. Xu Q. Tan Y. Wang G. (2015). Genomic characterization of a large panel of patient-derived hepatocellular carcinoma xenograft tumor models for preclinical development. *Oncotarget* 6 20160–20176. 10.18632/oncotarget.3969 26062443 PMC4652995

[B46] Gualdi R. Bossard P. Zheng M. Hamada Y. Coleman J. R. Zaret K. S. (1996). Hepatic specification of the gut endoderm in vitro: cell signaling and transcriptional control. *Genes Dev.* 10 1670–1682. 10.1101/gad.10.13.1670 8682297

[B47] Guan Y. Xu D. Garfin P. M. Ehmer U. Hurwitz M. Enns G. (2017). Human hepatic organoids for the analysis of human genetic diseases. *JCI Insight* 2:94954. 10.1172/jci.insight.94954 28878125 PMC5621886

[B48] Han S. Y. Dziedzic N. Gadue P. Keller G. M. Gouon-Evans V. (2011). An endothelial cell niche induces hepatic specification through dual repression of Wnt and notch signaling. *Stem Cells* 29 217–228. 10.1002/stem.576 21732480 PMC3437666

[B49] Hannan N. R. Segeritz C.-P. Touboul T. Vallier L. (2013). Production of hepatocyte-like cells from human pluripotent stem cells. *Nat. Protoc.* 8 430–437. 10.1038/nprot.2012.153 23424751 PMC3673228

[B50] Hidalgo M. Amant F. Biankin A. V. Budinska E. Byrne A. T. Caldas C. (2014). Patient-derived xenograft models: an emerging platform for translational cancer research. *Cancer Discov.* 4 998–1013. 10.1158/2159-8290.CD-14-0001 25185190 PMC4167608

[B51] Hindley C. J. Mastrogiovanni G. Huch M. (2014). The plastic liver: differentiated cells, stem cells, every cell? *J. Clin. Invest.* 124 5099–5102. 10.1172/JCI78372 25401467 PMC4348964

[B52] Hu H. Gehart H. Artegiani B. LÖpez-Iglesias C. Dekkers F. Basak O. (2018). Long-term expansion of functional mouse and human hepatocytes as 3D organoids. *Cell* 175 1591–1606.e19. 10.1016/j.cell.2018.11.013 30500538

[B53] Huch M. Bonfanti P. Boj S. F. Sato T. Loomans C. J. Van De Wetering M. (2013a). Unlimited in vitro expansion of adult bi-potent pancreas progenitors through the Lgr5/R-spondin axis. *EMBO J.* 32 2708–2721. 10.1038/emboj.2013.204 24045232 PMC3801438

[B54] Huch M. Dorrell C. Boj S. F. Van Es J. H. Li V. S. Van De Wetering M. (2013b). In vitro expansion of single Lgr5+ liver stem cells induced by Wnt-driven regeneration. *Nature* 494 247–250. 10.1038/nature11826 23354049 PMC3634804

[B55] Huch M. Gehart H. Van Boxtel R. Hamer K. Blokzijl F. Verstegen M. M. (2015). Long-term culture of genome-stable bipotent stem cells from adult human liver. *Cell* 160 299–312. 10.1016/j.cell.2014.11.050 25533785 PMC4313365

[B56] Huh D. Matthews B. D. Mammoto A. Montoya-Zavala M. Hsin H. Y. Ingber D. E. (2010). Reconstituting organ-level lung functions on a chip. *Science* 328 1662–1668. 10.1126/science.1188302 20576885 PMC8335790

[B57] Jin Y. Kim J. Lee J. S. Min S. Kim S. Ahn D.-H. (2018). Vascularized liver organoids generated using induced hepatic tissue and dynamic liver-specific microenvironment as a drug testing platform. *Adv. Funct. Mater.* 28:1801954 10.1002/adfm.201801954

[B58] Jung J. Zheng M. Goldfarb M. Zaret K. S. (1999). Initiation of mammalian liver development from endoderm by fibroblast growth factors. *Science* 284 1998–2003. 10.1126/science.284.5422.1998 10373120

[B59] Kamiya A. Kinoshita T. Ito Y. Matsui T. Morikawa Y. Senba E. (1999). Fetal liver development requires a paracrine action of oncostatin M through the gp130 signal transducer. *EMBO J.* 18 2127–2136. 10.1093/emboj/18.8.2127 10205167 PMC1171297

[B60] Kaplowitz N. (2005). Idiosyncratic drug hepatotoxicity. *Nat. Rev. Drug Discov.* 4 489–499. 10.1038/nrd1750 15931258

[B61] Kietzmann T. (2017). Metabolic zonation of the liver: the oxygen gradient revisited. *Redox Biol.* 11 622–630. 10.1016/j.redox.2017.01.012 28126520 PMC5257182

[B62] Kim J. B. (2005). Three-dimensional tissue culture models in cancer biology. *Semin. Cancer Biol.* 15 365–377. 10.1016/j.semcancer.2005.05.002 15975824

[B63] Koike H. Iwasawa K. Ouchi R. Maezawa M. Giesbrecht K. Saiki N. (2019). Modelling human hepato-biliary-pancreatic organogenesis from the foregut–midgut boundary. *Nature* 574 112–116. 10.1038/s41586-019-1598-0 31554966 PMC7643931

[B64] Kose E. Unal O. Bulbul S. Gunduz M. Haberle J. Arslan N. (2017). Identification of three novel mutations in fourteen patients with citrullinemia type 1. *Clin. Biochem.* 50 686–689. 10.1016/j.clinbiochem.2017.01.011 28132756

[B65] Kostadinova R. Boess F. Applegate D. Suter L. Weiser T. Singer T. (2013). A long-term three dimensional liver co-culture system for improved prediction of clinically relevant drug-induced hepatotoxicity. *Toxicol. Appl. Pharmacol.* 268 1–16. 10.1016/j.taap.2013.01.012 23352505

[B66] Koui Y. Kido T. Ito T. Oyama H. Chen S. W. Katou Y. (2017). An in vitro human liver model by iPSC-derived parenchymal and non-parenchymal cells. *Stem Cell Rep.* 9 490–498. 10.1016/j.stemcr.2017.06.010 28757162 PMC5549957

[B67] Kuwahara A. Ozone C. Nakano T. Saito K. Eiraku M. Sasai Y. (2015). Generation of a ciliary margin-like stem cell niche from self-organizing human retinal tissue. *Nat. Commun.* 6:6286. 10.1038/ncomms7286 25695148

[B68] Lancaster M. A. Knoblich J. A. (2014). Organogenesis in a dish: modeling development and disease using organoid technologies. *Science* 345:1247125. 10.1126/science.1247125 25035496

[B69] Lancaster M. A. Renner M. Martin C. A. Wenzel D. Bicknell L. S. Hurles M. E. (2013). Cerebral organoids model human brain development and microcephaly. *Nature* 501 373–379. 10.1038/nature12517 23995685 PMC3817409

[B70] Lee J. Bscke R. Tang P. C. Hartman B. H. Heller S. Koehler K. R. (2018). Hair follicle development in mouse pluripotent stem cell-derived skin organoids. *Cell Rep.* 22 242–254. 10.1016/j.celrep.2017.12.007 29298425 PMC5806130

[B71] Li L. Knutsdottir H. Hui K. Weiss M. J. He J. Philosophe B. (2019). Human primary liver cancer organoids reveal intratumor and interpatient drug response heterogeneity. *JCI Insight* [Epub ahead of print]. 30674722 10.1172/jci.insight.121490 PMC6413833

[B72] Lu H. Ma J. Yang Y. Shi W. Luo L. (2013). EpCAM is an endoderm-specific Wnt derepressor that licenses hepatic development. *Dev. Cell* 24 543–553. 10.1016/j.devcel.2013.01.021 23484855

[B73] Luo Y. Lou C. Zhang S. Zhu Z. Y. Xing Q. Z. Wang P. (2018). Three-dimensional hydrogel culture conditions promote the differentiation of human induced pluripotent stem cells into hepatocytes. *Cytotherapy* 20 95–107. 10.1016/j.jcyt.2017.08.008 28969895

[B74] Mansour A. A. Goncalves J. T. Bloyd C. W. Li H. Fernandes S. Quang D. (2018). An in vivo model of functional and vascularized human brain organoids. *Nat. Biotechnol.* 36 432–441. 10.1038/nbt.4127 29658944 PMC6331203

[B75] Mariotti V. Strazzabosco M. Fabris L. Calvisi D. F. (2018). Animal models of biliary injury and altered bile acid metabolism. *Biochim. Biophys. Acta Mol. Basis Dis.* 1864 1254–1261. 10.1016/j.bbadis.2017.06.027 28709963 PMC5764833

[B76] Massa S. Sakr M. A. Seo J. Bandaru P. Arneri A. Bersini S. (2017). Bioprinted 3D vascularized tissue model for drug toxicity analysis. *Biomicrofluidics* 11:044109. 10.1063/1.4994708 28852429 PMC5552405

[B77] Matano M. Date S. Shimokawa M. Takano A. Fujii M. Ohta Y. (2015). Modeling colorectal cancer using CRISPR-Cas9-mediated engineering of human intestinal organoids. *Nat. Med.* 21 256–262. 10.1038/nm.3802 25706875

[B78] Mederacke I. Dapito D. H. Affo S. Uchinami H. Schwabe R. F. (2015). High-yield and high-purity isolation of hepatic stellate cells from normal and fibrotic mouse livers. *Nat. Protoc.* 10 305–315. 10.1038/nprot.2015.017 25612230 PMC4681437

[B79] Mederacke I. Hsu C. C. Troeger J. S. Huebener P. Mu X. Dapito D. H. (2013). Fate tracing reveals hepatic stellate cells as dominant contributors to liver fibrosis independent of its aetiology. *Nat. Commun.* 4:2823. 10.1038/ncomms3823 24264436 PMC4059406

[B80] Meli L. Jordan E. T. Clark D. S. Linhardt R. J. Dordick J. S. (2012). Influence of a three-dimensional, microarray environment on human cell culture in drug screening systems. *Biomaterials* 33 9087–9096. 10.1016/j.biomaterials.2012.08.065 22998815 PMC3517800

[B81] Method of the Year 2017, (2018). Organoids. *Nat. Methods* 15:1.

[B82] Michalopoulos G. K. (2007). Liver regeneration. *J. Cell. Physiol.* 213 286–300. 10.1002/jcp.21172 17559071 PMC2701258

[B83] Michalopoulos G. K. (2010). Liver regeneration after partial hepatectomy: critical analysis of mechanistic dilemmas. *Am. J. Pathol.* 176 2–13. 10.2353/ajpath.2010.090675 20019184 PMC2797862

[B84] Mitaka T. Ooe H. (2010). Characterization of hepatic-organoid cultures. *Drug Metab. Rev.* 42 472–481. 10.3109/03602530903492020 20025558

[B85] Mun S. J. Ryu J. S. Lee M. O. Son Y. S. Oh S. J. Cho H. S. (2019). Generation of expandable human pluripotent stem cell-derived hepatocyte-like liver organoids. *J. Hepatol.* 71 970–985. 10.1016/j.jhep.2019.06.030 31299272

[B86] Nakano T. Ando S. Takata N. Kawada M. Muguruma K. Sekiguchi K. (2012). Self-formation of optic cups and storable stratified neural retina from human ESCs. *Cell Stem Cell* 10 771–785. 10.1016/j.stem.2012.05.009 22704518

[B87] Nie Y. Z. Zheng Y. W. Miyakawa K. Murata S. Zhang R. R. Sekine K. (2018). Recapitulation of hepatitis B virus-host interactions in liver organoids from human induced pluripotent stem cells. *EBioMedicine* 35 114–123. 10.1016/j.ebiom.2018.08.014 30120080 PMC6156717

[B88] Nuciforo S. Fofana I. Matter M. S. Blumer T. Calabrese D. Boldanova T. (2018). Organoid models of human liver cancers derived from tumor needle biopsies. *Cell Rep.* 24 1363–1376. 10.1016/j.celrep.2018.07.001 30067989 PMC6088153

[B89] Ogawa M. Ogawa S. Bear C. E. Ahmadi S. Chin S. Li B. (2015). Directed differentiation of cholangiocytes from human pluripotent stem cells. *Nat. Biotechnol.* 33 853–861. 10.1038/nbt.3294 26167630

[B90] Onitsuka I. Tanaka M. Miyajima A. (2010). Characterization and functional analyses of hepatic mesothelial cells in mouse liver development. *Gastroenterology* 138 1525–1535.e6. 10.1053/j.gastro.2009.12.059 20080099

[B91] Ootani A. Li X. Sangiorgi E. Ho Q. T. Ueno H. Toda S. (2009). Sustained in vitro intestinal epithelial culture within a Wnt-dependent stem cell niche. *Nat. Med.* 15 701–706. 10.1038/nm.1951 19398967 PMC2919216

[B92] Ouchi R. Togo S. Kimura M. Shinozawa T. Koido M. Koike H. (2019). Modeling steatohepatitis in humans with pluripotent stem cell-derived organoids. *Cell Metab* 30 374–384.e6. 10.1016/j.cmet.2019.05.007 31155493 PMC6687537

[B93] Pampaloni F. Reynaud E. G. Stelzer E. H. (2007). The third dimension bridges the gap between cell culture and live tissue. *Nat. Rev. Mol. Cell Biol.* 8 839–845. 10.1038/nrm2236 17684528

[B94] Pas̨ca A. M. Sloan S. A. Clarke L. E. Tian Y. Makinson C. D. Huber N. (2015). Functional cortical neurons and astrocytes from human pluripotent stem cells in 3D culture. *Nat. Methods* 12 671–678. 10.1038/nmeth.3415 26005811 PMC4489980

[B95] Peng W. C. Logan C. Y. Fish M. Anbarchian T. Aguisanda F. Alvarez-Varela A. (2018). Inflammatory cytokine TNFalpha promotes the long-term expansion of primary hepatocytes in 3D culture. *Cell* 175 1607–1619.e15. 10.1016/j.cell.2018.11.012 30500539 PMC6497386

[B96] Perea L. Coll M. Sancho-Bru P. (2015). “Assessment of liver fibrotic insults in vitro,” in *Protocols in In Vitro Hepatocyte Research*, eds Vinken M. Rogiers V. (Berlin: Springer), 391–401. 10.1007/978-1-4939-2074-7_30 26272160

[B97] Polini A. Prodanov L. Bhise N. S. Manoharan V. Dokmeci M. R. Khademhosseini A. (2014). Organs-on-a-chip: a new tool for drug discovery. *Expert Opin. Drug Discov.* 9 335–352. 10.1517/17460441.2014.886562 24620821

[B98] Potter S. S. (2018). Single-cell RNA sequencing for the study of development, physiology and disease. *Nat. Rev. Nephrol.* 14 479–492. 10.1038/s41581-018-0021-7 29789704 PMC6070143

[B99] Raven A. Lu W. Y. Man T. Y. Ferreira-Gonzalez S. O’duibhir E. Dwyer B. J. (2017). Cholangiocytes act as facultative liver stem cells during impaired hepatocyte regeneration. *Nature* 547 350–354. 10.1038/nature23015 28700576 PMC5522613

[B100] Rossi J. M. Dunn N. R. Hogan B. L. Zaret K. S. (2001). Distinct mesodermal signals, including BMPs from the septum transversum mesenchyme, are required in combination for hepatogenesis from the endoderm. *Genes Dev.* 15 1998–2009. 10.1101/gad.904601 11485993 PMC312750

[B101] Rowe R. G. Daley G. Q. (2019). Induced pluripotent stem cells in disease modelling and drug discovery. *Nat. Rev. Genet.* 20 377–388. 10.1038/s41576-019-0100-z 30737492 PMC6584039

[B102] Sampaziotis F. De Brito M. C. Geti I. Bertero A. Hannan N. R. F. Vallier L. (2017). Directed differentiation of human induced pluripotent stem cells into functional cholangiocyte-like cells. *Nat. Protoc.* 12 814–827. 10.1038/nprot.2017.011 28333915 PMC5467722

[B103] Sampaziotis F. De Brito M. C. Madrigal P. Bertero A. Saeb-Parsy K. Soares F. A. C. (2015). Cholangiocytes derived from human induced pluripotent stem cells for disease modeling and drug validation. *Nat. Biotechnol.* 33 845–852. 10.1038/nbt.3275 26167629 PMC4768345

[B104] Sato T. Vries R. G. Snippert H. J. Van De Wetering M. Barker N. Stange D. E. (2009). Single Lgr5 stem cells build crypt-villus structures in vitro without a mesenchymal niche. *Nature* 459 262–265. 10.1038/nature07935 19329995

[B105] Schaub J. R. Malato Y. Gormond C. Willenbring H. (2014). Evidence against a stem cell origin of new hepatocytes in a common mouse model of chronic liver injury. *Cell Rep.* 8 933–939. 10.1016/j.celrep.2014.07.003 25131204 PMC4376310

[B106] Schepers A. Li C. Chhabra A. Seney B. T. Bhatia S. (2016). Engineering a perfusable 3D human liver platform from iPS cells. *Lab Chip* 16 2644–2653. 10.1039/c6lc00598e 27296616 PMC5318999

[B107] Schmelzer E. Zhang L. Bruce A. Wauthier E. Ludlow J. Yao H. L. (2007). Human hepatic stem cells from fetal and postnatal donors. *J. Exp. Med.* 204 1973–1987. 10.1084/jem.20061603 17664288 PMC2118675

[B108] Scholten D. Osterreicher C. H. Scholten A. Iwaisako K. Gu G. Brenner D. A. (2010). Genetic labeling does not detect epithelial-to-mesenchymal transition of cholangiocytes in liver fibrosis in mice. *Gastroenterology* 139 987–998. 10.1053/j.gastro.2010.05.005 20546735 PMC2930026

[B109] Schutgens F. Rookmaaker M. B. Margaritis T. Rios A. Ammerlaan C. Jansen J. (2019). Tubuloids derived from human adult kidney and urine for personalized disease modeling. *Nat. Biotechnol.* 37 303–313. 10.1038/s41587-019-0048-8 30833775

[B110] Shin S. Upadhyay N. Greenbaum L. E. Kaestner K. H. (2015). Ablation of Foxl1-Cre-labeled hepatic progenitor cells and their descendants impairs recovery of mice from liver injury. *Gastroenterology* 148 192–202.e3. 10.1053/j.gastro.2014.09.039 25286440 PMC4387775

[B111] Si-Tayeb K. Lemaigre F. P. Duncan S. A. (2010a). Organogenesis and development of the liver. *Dev. Cell* 18 175–189. 10.1016/j.devcel.2010.01.011 20159590

[B112] Si-Tayeb K. Noto F. K. Nagaoka M. Li J. Battle M. A. Duris C. (2010b). Highly efficient generation of human hepatocyte-like cells from induced pluripotent stem cells. *Hepatology* 51 297–305. 10.1002/hep.23354 19998274 PMC2946078

[B113] Spence J. R. Mayhew C. N. Rankin S. A. Kuhar M. F. Vallance J. E. Tolle K. (2011). Directed differentiation of human pluripotent stem cells into intestinal tissue in vitro. *Nature* 470 105–109. 10.1038/nature09691 21151107 PMC3033971

[B114] Stanger B. Z. (2015). Cellular homeostasis and repair in the mammalian liver. *Annu. Rev. Physiol.* 77 179–200. 10.1146/annurev-physiol-021113-170255 25668020 PMC5830102

[B115] Sun L. Wang Y. Cen J. Ma X. Cui L. Qiu Z. (2019). Modelling liver cancer initiation with organoids derived from directly reprogrammed human hepatocytes. *Nat. Cell Biol.* 21 1015–1026. 10.1038/s41556-019-0359-5 31332348

[B116] Takahashi K. Tanabe K. Ohnuki M. Narita M. Ichisaka T. Tomoda K. (2007). Induction of pluripotent stem cells from adult human fibroblasts by defined factors. *Cell* 131 861–872. 10.1016/j.cell.2007.11.019 18035408

[B117] Takasato M. Er P. X. Chiu H. S. Maier B. Baillie G. J. Ferguson C. (2015). Kidney organoids from human iPS cells contain multiple lineages and model human nephrogenesis. *Nature* 526 564–568. 10.1038/nature15695 26444236

[B118] Takayama K. Mitani S. Nagamoto Y. Sakurai F. Tachibana M. Taniguchi Y. (2016). Laminin 411 and 511 promote the cholangiocyte differentiation of human induced pluripotent stem cells. *Biochem. Biophys. Res. Commun.* 474 91–96. 10.1016/j.bbrc.2016.04.075 27103433

[B119] Takebe T. Sekine K. Enomura M. Koike H. Kimura M. Ogaeri T. (2013). Vascularized and functional human liver from an iPSC-derived organ bud transplant. *Nature* 499 481–484. 10.1038/nature12271 23823721

[B120] Tanaka M. Okabe M. Suzuki K. Kamiya Y. Tsukahara Y. Saito S. (2009). Mouse hepatoblasts at distinct developmental stages are characterized by expression of EpCAM and DLK1: drastic change of EpCAM expression during liver development. *Mech. Dev.* 126 665–676. 10.1016/j.mod.2009.06.939 19527784

[B121] Tremblay K. D. Zaret K. S. (2005). Distinct populations of endoderm cells converge to generate the embryonic liver bud and ventral foregut tissues. *Dev. Biol.* 280 87–99. 10.1016/j.ydbio.2005.01.003 15766750

[B122] Turnpenny P. D. Ellard S. (2012). Alagille syndrome: pathogenesis, diagnosis and management. *Eur. J. Hum. Genet.* 20 251–257. 10.1038/ejhg.2011.181 21934706 PMC3283172

[B123] Underhill G. H. Khetani S. R. (2018). Bioengineered liver models for drug testing and cell differentiation studies. *Cell. Mol. Gastroenterol. Hepatol.* 5 426–439.e1. 10.1016/j.jcmgh.2017.11.012 29675458 PMC5904032

[B124] van de Wetering M. Francies H. E. Francis J. M. Bounova G. Iorio F. Pronk A. (2015). Prospective derivation of a living organoid biobank of colorectal cancer patients. *Cell* 161 933–945. 10.1016/j.cell.2015.03.053 25957691 PMC6428276

[B125] van den Berg C. W. Ritsma L. Avramut M. C. Wiersma L. E. Van Den Berg B. M. Leuning D. G. (2018). Renal subcapsular transplantation of PSC-derived kidney organoids induces Neo-vasculogenesis and significant glomerular and tubular maturation in vivo. *Stem Cell Rep.* 10 751–765. 10.1016/j.stemcr.2018.01.041 29503086 PMC5918682

[B126] Vyas D. Baptista P. M. Brovold M. Moran E. Gaston B. Booth C. (2017). Self-assembled liver organoids recapitulate hepatobiliary organogenesis in vitro. *Hepatology* 67 750–761. 10.1002/hep.29483 28834615 PMC5825235

[B127] Wang B. Zhao L. Fish M. Logan C. Y. Nusse R. (2015). Self-renewing diploid Axin2(+) cells fuel homeostatic renewal of the liver. *Nature* 524 180–185. 10.1038/nature14863 26245375 PMC4589224

[B128] Wang Y. Wang H. Deng P. Chen W. Guo Y. Tao T. (2018). In situ differentiation and generation of functional liver organoids from human iPSCs in a 3D perfusable chip system. *Lab Chip* 18 3606–3616. 10.1039/c8lc00869h 30357207

[B129] Wells R. G. (2014). The portal fibroblast: not just a poor man’s stellate cell. *Gastroenterology* 147 41–47. 10.1053/j.gastro.2014.05.001 24814904 PMC4090086

[B130] Wimmer R. A. Leopoldi A. Aichinger M. Wick N. Hantusch B. Novatchkova M. (2019). Human blood vessel organoids as a model of diabetic vasculopathy. *Nature* 565 505–510. 10.1038/s41586-018-0858-8 30651639 PMC7116578

[B131] Wu F. Wu D. Ren Y. Huang Y. Feng B. Zhao N. (2019). Generation of hepatobiliary organoids from human induced pluripotent stem cells. *J. Hepatol.* 70 1145–1158. 10.1016/j.jhep.2018.12.028 30630011

[B132] Yanger K. Knigin D. Zong Y. W. Maggs L. Gu G. Q. Akiyama H. (2014). Adult hepatocytes are generated by self-duplication rather than stem cell differentiation. *Cell Stem Cell* 15 340–349. 10.1016/j.stem.2014.06.003 25130492 PMC4505916

[B133] Zaret K. S. (2002). Regulatory phases of early liver development: paradigms of organogenesis. *Nat. Rev. Genet.* 3 499–512. 10.1038/nrg837 12094228

[B134] Zhang Y. S. Aleman J. Shin S. R. Kilic T. Kim D. Mousavi Shaegh S. A. (2017a). Multisensor-integrated organs-on-chips platform for automated and continual in situ monitoring of organoid behaviors. *Proc. Natl. Acad. Sci. U.S.A.* 114 E2293–E2302. 10.1073/pnas.1612906114 28265064 PMC5373350

[B135] Zhang Y. S. Yue K. Aleman J. Moghaddam K. M. Bakht S. M. Yang J. (2017b). 3D bioprinting for tissue and organ fabrication. *Ann. Biomed. Eng.* 45 148–163. 10.1007/s10439-016-1612-8 27126775 PMC5085899

[B136] Zhou W. C. Zhang Q. B. Qiao L. (2014). Pathogenesis of liver cirrhosis. *World J. Gastroenterol.* 20 7312–7324. 10.3748/wjg.v20.i23.7312 24966602 PMC4064077

